# *KRAS* mutation analysis of single circulating tumor cells from patients with metastatic colorectal cancer

**DOI:** 10.1186/s12885-017-3305-6

**Published:** 2017-05-03

**Authors:** Yuurin Kondo, Kazuhiko Hayashi, Kazuyuki Kawakami, Yukari Miwa, Hiroshi Hayashi, Masakazu Yamamoto

**Affiliations:** 1 Department of Chemotherapy and Palliative Care, Tokyo Women’s Medical University, 8-1 Kawada-chyo, Shinjuku-ku, Tokyo, 162-8666 Japan; 2 Research & Development Department, SRL, Inc., Shinjuku, Japan; 3 Department of Surgery, Institute of Gastroenterology, Tokyo Women’s Medical University, Shinjuku, Japan

**Keywords:** Circulating tumor cells, Mutation analysis, *KRAS*, Single cell analysis, Heterogeneity

## Abstract

**Background:**

The molecular profiles of tumors may inform the selection of appropriate targeted therapies. Circulating tumor cells (CTCs) reflect the real-time status of tumor genotypes. CTCs exhibit high genetic heterogeneity within a patient; accordingly, the analysis of individual CTCs, including their heterogeneity, may enable more precise treatments. We analyzed *KRAS* mutations in single CTCs from patients with metastatic colorectal cancer (mCRC) using a new single-cell picking system.

**Methods:**

Blood samples were obtained from 61 patients with mCRC. CTCs were enriched and fluorescently labeled using the CellSearch® System. They were recovered using the single-cell picking system based on the fluorescence intensity of marker dyes. Single CTCs and tumor tissue samples were examined for mutations in codons 12 and 13 of the *KRAS* gene.

**Results:**

CTCs were detected in 27 of 61 patients with mCRC. We isolated at least two CTCs from 15 of 27 patients. *KRAS* genotype was evaluated in a total of 284 CTCs from 11 patients, and 15 cells with mutations were identified in four patients. In 10 of 11 patients, the *KRAS* status was the same in the primary tumor and CTCs. In one patient, the *KRAS* status was discordant between the primary tumor and CTCs. In two patients, different *KRAS* mutations were found among individual CTCs.

**Conclusions:**

We successfully isolated single CTCs and detected *KRAS* mutations in individual cells from clinical samples using a novel application of single-cell isolation system. Using the system, we detected CTC heterozygosity and heterogeneity in *KRAS* status among CTCs within a patient and between CTCs and tumor tissues.

## Background

Colorectal cancer (CRC) is one of the leading causes of cancer deaths worldwide. Recently, the use of new antitumor agents for metastatic CRC (mCRC), such as epidermal growth factor receptor-targeted monoclonal antibodies (anti-EGFR), has significantly improved the treatment of colorectal disease [1, 2].

*KRAS* mutations are present in 30–40% of CRC patients [3]. Activating mutations in *KRAS* are responsible for anti-EGFR therapy resistance in mCRC; accordingly, *KRAS* genotyping is recommended before EGFR-targeted therapies are administered (e.g., cetuximab and panitumumab) [4]. Although *KRAS* is a negative predictive marker, not all patients with wild-type *KRAS* in tumor cells respond to EGFR-targeted therapies. *KRAS* genotype may not be an accurate predictor of treatment response owing to genetic differences between primary and metastatic tumors.

Several studies have shown that distant metastases can have unique genetic alterations that are different from those in the primary tumor [5, 6]. In addition, acquired resistance is partly achieved by the selection of pre-existing minor subclones harboring mutations that confer resistance to targeted therapy [7, 8]. Primary tumor specimens are not always representative of metastases, which can occur many years after resection of the primary tumor [9, 10]. Characterization of metastatic sites may provide more important information than characterization of primary tumors with respect to guiding targeted therapies [11]. However, invasive biopsies of metastatic sites are not always feasible and repeated testing for real-time surveillance is often difficult.

To overcome the abovementioned problems, circulating tumor cells (CTCs), which can be analyzed clinically by “liquid biopsy,” may be useful for the noninvasive characterization of tumors. These cells reflect subpopulations of primary and/or metastatic tumor cells and are accessible by blood collection [12]. The number of CTCs is correlated with prognosis in several tumor types, such as breast, prostate, and colorectal cancers [13–15]. Monitoring alterations in CTC number during anticancer treatment not only improves prognostic prediction, but also provides information regarding therapy response [14–20]. In addition to enumeration, the molecular characterization of CTCs is important for therapeutic decision-making [21].

Among other challenges with respect to CTC characterization, the isolation of pure CTCs that are not contaminated with leukocytes is still difficult owing to their rarity in peripheral blood [12]. Several studies have detected heterogeneity among CTCs at the single cell level [22, 23]. This suggests the importance of analyzing CTCs at the single-cell level for accurate tumor profiling. However, genetic heterogeneity has not been incorporated into clinical treatments.

Here, we demonstrated the feasibility of detecting *KRAS* mutations in single CTCs isolated from mCRC patients in a novel application of an automated single-cell isolation system to identify individual cancer cells. Our objective was to analyze high-purity CTCs using this cell recovery system and to evaluate the discordance in *KRAS* status between primary tumors and CTCs as well as variation among CTCs.

## Methods

### Ethics and consent statement

This study was approved by the ethical committee of Tokyo Women’s Medical University (approval number, 247) and all patients provided written informed consent prior to participation in the study. All participants in this study provided written informed consent for the publication of their clinical details.

### Cell lines

The H1975 human lung cancer cell line containing *EGFR* mutations was obtained from the ATCC Cell Bank (Manassas, VA, USA) and was used for cell-recovery experiments. The A549 human lung cancer cell line containing *KRAS* mutations was obtained from the ATCC Cell Bank and was used for blood spiking experiments. H1975 was cultured in RPMI-1640 medium containing 10% fetal bovine serum (both from Thermo fisher scientific, Waltham, MA, USA) in a humidified 5% CO_2_ incubator at 37 °C. A549 was cultured in F-12 K medium (Thermo fisher scientific) containing 10% fetal bovine serum in a humidified 5% CO_2_ incubator at 37 °C.

### Tumor cell enrichment, staining, and enumeration

The enrichment and enumeration of tumor cells from whole blood were performed using the FDA-approved CellSearch® System (Janssen Diagnostics, Raritan, NJ, USA). First, 7.5 mL of the whole blood sample was processed using the CellSearch® CTC Kit (Janssen Diagnostics). In this assay, EpCAM-based immunomagnetically enriched cells were fluorescently counterlabeled with DAPI to stain nuclei, phycoerythrin (PE)-conjugated antibodies directed against cytokeratins 8/18/19, and allophycocyanin (APC)-conjugated antibodies directed against CD45 to stain the remaining WBCs**.** After enrichment, isolated fluorescently labeled cells were resuspended in a MagNest^Ⓡ^ Cartridge Holder (Janssen Diagnostics) and analyzed (i.e., identified and enumerated) using the CellTracks Analyzer II^Ⓡ^ (Janssen Diagnostics) according to the manufacturer’s instructions.

### Single CTC isolation

To isolate single cells, an automated single-cell isolation system was used, i.e., the ASONECell Picking System (ASONE, Osaka, Japan), to identify individual cancer cells based on staining [24].

**Fig. 1:**
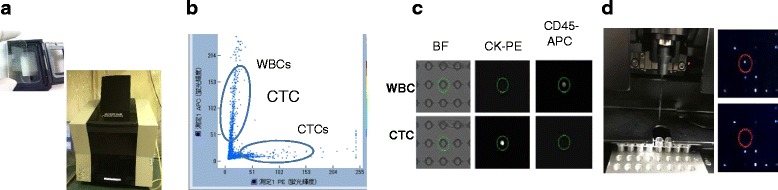
Summary of the ASONECell Picking System. **a** Fluorescently labeled cells are loaded in a microchamber array and sorted by the machine. **b** Scatter plot of mean fluorescence intensities for CK-PE (*x*-axis) and CD45-APC (*y*-axis) staining. **c** Bright-field, PE, and APC channel images of peripheral blood mononuclear cells (PBMCs) and circulating tumor cells (CTCs). CTCs can be distinguished from contaminated leukocytes by combining the fluorescence filters. **d** Cells marked with a *red circle* are automatically collected with a glass capillary

Each CTC-enriched sample was recovered from the CellSearch® cartridge and manually loaded onto the microchamber array chip (84,640 wells of 30-μm diameter, 196,000 wells of 20-μm diameter). The fluorescently labeled cells were introduced into each well of the microchamber by centrifugation (2 repetitions of acceleration at 200 rpm for 1 min by plate centrifugation). After loading the microchamber array into the single-cell picking system, the fluorescence intensity of each cell was scanned and analyzed using a computer with a robot. Cells of interest were marked according to PE, APC, and DAPI fluorescence intensity. Marked cells were automatically collected with a glass capillary attached to the micromanipulator of the robot. Each cell was transferred and recovered in 10 μL of PBS in a 200-μL PCR tube. The samples were dried completely then stored in a deep freezer at −80 °C until use. Representative images obtained using the ASONECell Picking System are shown in Fig. 1.

### Evaluation of cell collection using the new single cell picking application

A solution of H1975 cells stained with Cell Tracker™ Green (Thermo fisher scientific) was loaded on the single-cell picking system and single cells were collected and added to individual wells of a 96-well microplate. The existence of a single cell in each well was confirmed by fluorescent microscopy. To quantify tumor cells identified using the single-cell picking system, approximately 1500 or a small number of (2–25) A549 cells were spiked into 7.5 mL of whole blood from a healthy donor (HD), which was collected in a CellSave Preservative Tube (Janssen Diagnostics). A549 cells spiked in HD blood were processed using the CellSearch® CTC Kit (Janssen Diagnostics), and A549 cell counts were determined using the CellTracks Analyzer II® (Janssen Diagnostics). Enriched cells were loaded onto the single-cell picking system and re-counted. CTC counts obtained by CellSearch® and the single-cell picking system were compared. When a small number of cells, i.e., A549 cells, were spiked, single cells were recovered and the recovery rate was calculated.

### Preclinical validation of single cell *KRAS* mutation detection using the A549 cell line

To assess the feasibility of using recovered cells for downstream analyses, a known number of A549 cells was added to 7.5 mL of peripheral blood obtained from an HD, collected in a CellSave Preservative tube, and enriched using the CellSearch® system. Then, single cells were recovered into individual PCR tubes using the single-cell picking system. A total of 24 recovered A549 cells were subjected to *KRAS* gene-specific amplification after cell lysis with proteinase K (Takara Bio, Kusatsu, Japan) and sodium dodecyl sulfate in individual PCR tubes as previously demonstrated [25]. The DNA from single cell was subjected to *KRAS* gene-specific amplification and sequenced using the same protocol as that used for CTCs described below. Nine single WBCs isolated from blood samples also served as wild type control for sequencing.

### Patient enrolment and tissue and sample collection

The study included 61 patients who had mCRC and underwent various anticancer therapies at the Department of Chemotherapy and Palliative Care or the Department of Surgery, Institute of Gastroenterology, Tokyo Women’s Medical University Hospital. Paraffin-embedded or fresh frozen sections collected from primary tumors were used for *KRAS* characterization. For each patient, two 10-mL blood samples were drawn into CellSave Preservative tubes or EDTA tubes for CTC enrichment, enumeration, and a mutation analysis. Blood samples were processed within 72 h of collection.

### *KRAS* mutation analysis

A total of 284 single CTCs were analyzed by direct sequencing of the *KRAS* gene. Sequencing was performed using DNA isolated from CTCs directly or following whole-genome amplification (WGA). For the former analysis, a total of 107 single CTCs were subjected to *KRAS* gene-specific amplification after cell lysis with proteinase K and sodium dodecyl sulfate. The following nested PCR primers for *KRAS* codons 12 and 13 were designed using Primer3: outer primers, forward 5′-AAGGTACTGGTGGAGTATTTG-3′ and reverse 5′-GTACTCATGAAAATGGTGAGA-3′; inner primers, forward 5′-ATTATAAGGCCTGCTGAAAATGAGTGA-3′ and reverse 5′-ATATGCATATTAAAACAAGATTTACCTCTA-3′. The reaction was amplified for 40 cycles at 94, 59, and 72 °C for 30 s per cycle for each temperature. The remaining 177 single CTCs were first subjected to WGA using the Ampli1™ WGA Kit (Silicon Biosystems, Bologna, Italy) according to the manufacturer’s instructions. They were then subjected to *KRAS* gene-specific amplification using the following primers: forward 5′-CCTTATGTGTAGCATGTTCTAATATAG-3′ and reverse 5′-CTATTGTTGGATCATATTCGTCCAC-3′.

Amplified DNA from CTCs was used for direct sequencing of *KRAS*. PCR products were sequenced using the Big Dye Terminator 3.1 Cycle Sequencing Kit (Applied Biosystems, Foster City, CA, USA). The sequencing reaction was analyzed using a 3130xl Genetic Analyzer (Applied Biosystems).

DNA from primary tumor tissue was extracted using the FFPE Tissue Kit (Qiagen, Hilden, Germany), subjected to *KRAS* gene-specific amplification, and sequenced using the same protocol as that used for CTCs.

## Results

### Evaluation of single-cell collection using the single-cell picking system

**Fig. 2:**
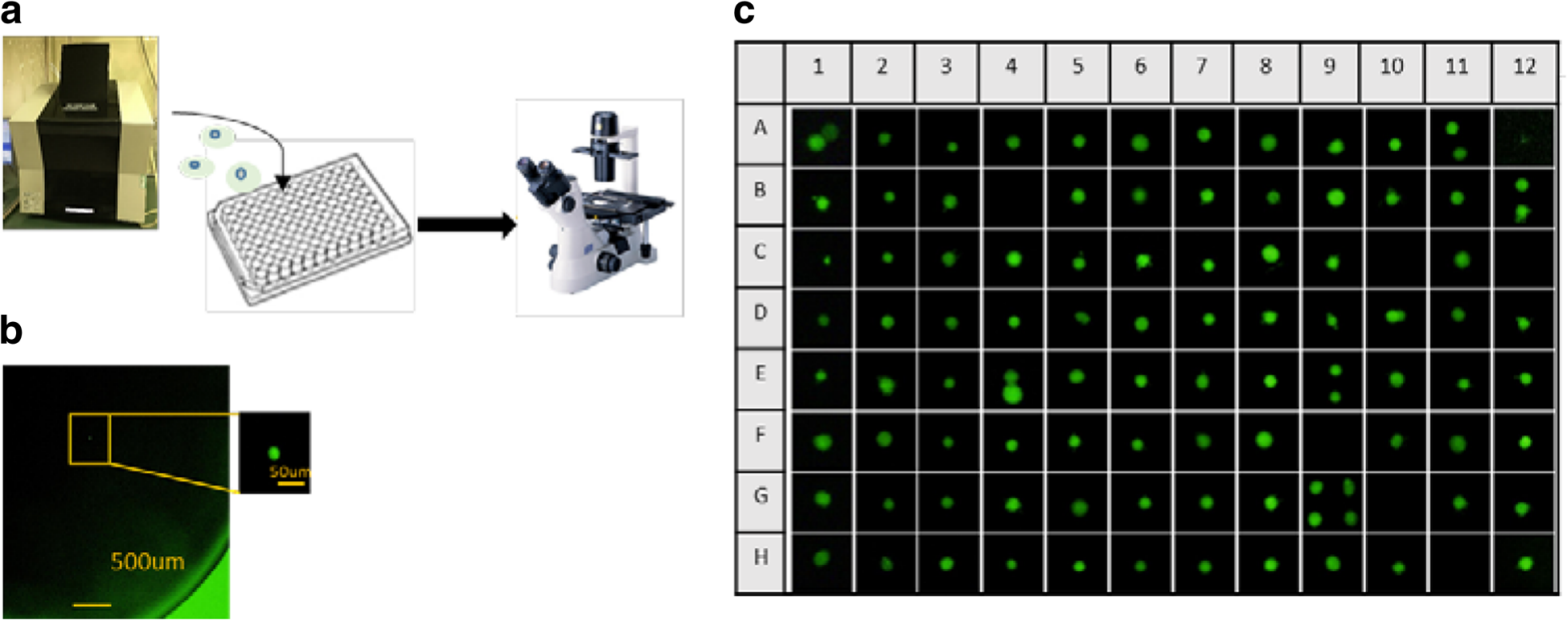
Single-cell collection. **a** H1975 cells stained by Cell Tracker Green were loaded onto the single-cell and collected into 96-well microplate (200uL PBS/well). **b** The picture of the isolated single cell confirmed by fluorescent microscopy. **c** Images of the recovered cells in each well of 96-well microplate. In 84 wells, isolation of single-cell was succeeded. In 12 wells, isolation was failed. In six of 12 wells, more than one cell was collected. Isolation success yield was 87.5% (84/96)

To quantify the rate of tumor cell recovery using the single-cell picking system, fluorescently labeled H1975 cells were loaded onto the single-cell picking system and collected individually in wells of a 96-well microplate. Single cells were found in 84 out of 96 wells using fluorescence microscopy, for an isolation success rate of 87.5% (Fig. 2). We next assessed the recovery rate of single CTCs from CellSearch® system. Enriched cells in CellSearch® cartridges were loaded into the single-cell picking system and analyzed. The results of eight independent experiments are summarized in Table 1. In a comparative cell identification analysis, 73.4% of the total cells detected using the CellSearch system were observed using the single-cell picking system after loading into the microchamber, on average. We examined the recovery rate using serial dilutions to obtain a more clinically relevant range (2–25 cells). The results are shown in Table 2. The recovery rate was 70.8%, on average (range 38.5–100%).

**Table 1:** Comparison of tumor cell counts obtained using CellSearch and the ASONECell Picking System

*n* = 8	CellSearch® (cells)	ASONECell Picking System (cells)	Re-identification rate (%) ASONECell/ CellSearch®
1	1634	1072	65.6%
2	1692	1258	74.3%
3	1674	1430	85.4%
4	1827	1463	80.1%
5	1874	1335	71.2%
6	1927	1369	71%
7	1964	1324	67.4%
8	1783	1289	72.3%
Average	1797	1318	73.4%

**Table 2:** Re-identification rate and recovery rate for a small number of cells (2–25 cells)

*n* = 9	CellSearch® Count (cells)	ASONECell		Re-identification rate^a^ (%)	Recovery rate^b^ (%)
		Count (cells)	Pick up (cells)		
1	2	1	1	50%	50%
2	2	2	2	100%	100%
3	4	4	4	100%	100%
4	8	7	5	87.5%	62.5%
5	13	6	5	46.2%	38.5%
6	19	15	14	78.9%	73.7%
7	21	13	11	61.9%	52.4%
8	25	21	18	84%	72%
9	25	23	22	92%	88%
Average	13.2	9.7	9.1	77.8%	70.8%

^a^Re-identification rate, the number of cells counted using CellSearch® divided by the number of cells re-counted using ASONECell Picking system

^b^Recovery rate, the number of cells counted using CellSearch® divided by the number of cells picked up using ASONEcell Picking system

### Preclinical validation of single cell *KRAS* mutation detection using the A549 cell line

After CellSearch® enrichment, 24 single A549 cells were recovered by the single-cell picking system and subjected to *KRAS* gene-specific amplification. The A549 cell line harbors homozygous *KRAS* mutation (G12S). Codons 12 and 13 of the *KRAS* gene were sequenced in all sorted cells. In all recovered single A549 cells, *KRAS* mutation of codon 12 was detected. In 21 of the 24 single A549 cells, the known original homozygous mutation was detected. In the remaining three single A549 cells, the wild-type *KRAS* allele was detected by sequencing, in addition to the mutant allele (i.e., the samples were heterozygous). This may be explained by contamination with HD blood. Nine single WBCs isolated from HD blood sample were confirmed the expected wild-type genotype.

### Patient characteristics

Sixty-one mCRC patients were enrolled in the study. The patient characteristics, including the number of CTCs based on CellSearch®, are listed in Table 3. CTCs (≥1) were detected in 27 out of 61 (44.3%) patients. The range of CTC counts in the CTC-positive patient group (CTC ≥ 1) was 1 to 105 cells.

**Table 3:** Patient characteristics according to CTC number assessed by CellSearch

Patients’ characteristics	CTC = 0	CTC ≥ 1	Total (%)
	*n* = 34	*n* = 27	*n* = 61
Age			
Median	69	63	67
(range)	(34–80)	(36–82)	(34–82)
Site of primary tumor			
Right hemicolon	14	9	23 (38%)
Left hemicolon	7	7	14 (23%)
Rectum	13	10	23 (38%)
Other	0	1	1 (1%)
Site of metastasis			
Liver only	14	10	24 (40%)
Others	20	17	37 (60%)
Disease status			
Primary	11	12	23 (38%)
Recurrence	23	15	38 (62%)
*KRAS* status in primary tissue			
Wild-type	21	14	35 (57%)
Mutant	9	10	19 (31%)
Unknown	4	3	7 (12%)

In the CTC-negative patient group (CTC = 0), a *KRAS* mutation was found in 9 out of 34 (25%) patients. In the CTC-positive patient group, the mutation was found in 10 out of 27 (37%) patients. The presence of CTCs was not related to clinical characteristics.

### Evaluation of CTCs in clinical samples

**Fig. 3:**
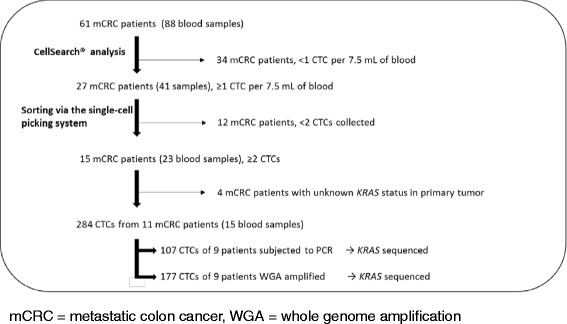
Sample flowchart

Eighty-eight blood samples from 61 patients were analyzed using CellSearch®; the full analysis is summarized in the sample flowchart shown in Fig. 3. Samples obtained from 27 patients (44.3%) for whom at least one CTC was detected using CellSearch® were selected for sorting by the single-cell picking system. For 15 (24.6%) of these 27 patients, at least two single CTCs were recovered by the single-cell picking system.

Single CTCs recovered from 11 (18%) patients from whom primary tumor samples were available were evaluated to determine the *KRAS* genotype; primary tumor samples were also sequenced in these cases.

### *KRAS* mutational status of single CTCs determined by PCR

A total of 284 single CTCs were recovered from 11 mCRC patients; 107 single CTCs from nine patients were subjected to direct *KRAS* gene-specific amplification and 77 were successfully sequenced (median percentage of sequenced CTCs per patient, 70%; range, 20–100%; Table 4, left panel). Sequencing failure may reflect cell loss during sample manipulation or PCR amplification failure.

**Table 4:** *KRAS* mutation analysis in single CTCs and primary tissue

Pt ID	Blood Sample ID	CTC												Primary tumor
		PCR						WGA						
		Number of samples analyzed	Number of successful sequences	Sequence success rate (%)	*KRAS* status			Number of samples analyzed	Number of successful sequences	Sequence success rate (%)	*KRAS* status			
					Number of wild-type cells	Number of mutant cells					Number of wild-type cells	Number of mutant cells		
I	#43	12	12	100%	12	0	wild	9	7	77.8%	7	0	wild	wild
	#46	12	12	100%	12	0	wild	18	16	88.9%	16	0	wild	
II	#44	2	2	100%	2	0	wild	-	-	-	-	-	-	wild
III	#82	3	3	100%	1	2	p.G12A		-	-	-	-	-	p.G12A
VI	#92	10	7	70%	7	0	wild	-	-	-	-	-	-	wild
	#96	12	8	66.7%	8	0	wild	1	1	100%	1	0	wild	
V	#95	-	-	-	-	-	-	6	6	100%	6	0	wild	wild
	#97	10	6	60%	6	0	wild	16	14	87.5%	14	0	wild	
	#142	-	-	-	-	-	-	4	4	100%	4	0	wild	
VI	#98	10	5	50%	3	2	p.G12D	8	2	25%	2	0	wild	p.G12D
	#99	12	8	66.7%	4	4	p.G12D	74	73	98.6%	70	3	p.G12D	
VII	#114	5	1	20%	1	0	wild	5	3	60%	3	0	wild	wild
VIII	#126	-	-	-	-	-	-	4	3	75%	3	0	wild	wild
	#129	-	-	-	-	-	-	7	6	85.7%	6	0	wild	
IX	#130	6	5	83.3%	5	0	wild	8	8	100%	7	1	p.G13D	wild
	#131	6	2	33.3%	1	1	p.G13D	13	7	53.8%	6	1	p.G12D	
X	#135	-	-	-	-	-	-	2	2	100%	2	0	wild	wild
XI	#137	7	6	85.7%	5	1	p.G12D	2	1	50%	1	0	wild	p.G12D
	Total	107	77	72%				177	153	85.9%				

CTCs from five of nine patients had wild-type *KRAS* at codons 12 and 13. Ten CTCs from the remaining four patients (Patients III, VI, IX, and XI) contained mutations in the *KRAS* gene.

**Fig. 4:**
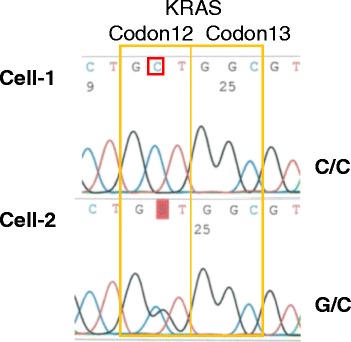
*KRAS* mutations in single CTCs from Patient III. Direct sequencing results for *KRAS* codons 12 and 13; the mutation in codon 12 was homozygosis in Cell-1 and heterozygosis in Cell-2

For Patient III, a c.35G > C (p.G12A) mutation in codon 12 of the *KRAS* gene was detected in two of three CTCs. In one CTC, the mutation was homozygous, while it was heterozygous in the other (Fig. 4). Thus, CTCs exhibited genetic heterogeneity at the single-cell level and showed the potential for loss of heterozygosity of the wild-type allele.

For Patient VI, sample #98 contained a c.35G > A (p.G12D) mutation in codon 12 of the *KRAS* gene in two of five CTCs and sample #99 had the same mutation in four of eight CTCs. For Patient IX, sample #130 had the wild-type *KRAS* genotype for all five analyzed CTCs and sample #131 contained a c.38G > A (p.G13D) mutation in codon 12 in one of two CTCs. For Patient XI, the c.35G > A (p.G12D) mutation in codon12 was detected in one of six CTCs.

### *KRAS* mutational status of single CTCs subjected to WGA

The remaining 177 single CTCs from nine patients were subjected to WGA. *KRAS* of 153 CTCs was successfully sequenced (median percentage of sequenced CTCs per patient, 85.9%; range, 25–100%; Table 4, right panel). Sequencing failure may have been caused by cell loss during sample manipulation, the WGA reaction, or PCR amplification failure.

CTCs from seven of nine patients were wild type for *KRAS* codons 12 and 13. Five CTCs from the remaining two patients (Patient VI and IX) contained mutations in the *KRAS* gene.

For patient VI, sample #98 did not have a mutation in *KRAS* codons 12 and 13 in the two analyzed CTCs and sample #99 contained a c.35G > A (p.G12D) mutation in codon 12 in three of 73 CTCs. For patient IX, two serial blood samples contained different mutations. Sample #130 showed a c.38G > A (p.G13D) mutation in codon 13 in one of eight CTCs and sample #131 contained a c.35G > A (p.G12D) mutation in codon 12 in one of seven CTCs.

### *KRAS* mutational status of primary tissues compared with CTCs

Primary tumor tissues were available for 11 patients. The *KRAS* mutation status for each of these samples is summarized in Table 4. Wild-type *KRAS* was detected in eight of 11 samples, while mutant *KRAS* was detected in three primary tumor samples. In seven of 11 patients, both CTCs and primary tissues were wild type for codons 12 and 13 of the *KRAS* gene. In one patient (Patient III), both CTCs and primary tissues showed the same mutation in the *KRAS* gene. In the remaining three patients (Patient VI, IX, and XI), there was discordance between the *KRAS* mutational status of primary tumor tissues and CTCs.

## Discussion

In this study, we evaluated the feasibility of detecting *KRAS* mutations in single CTCs isolated from mCRC patients using the ASONECell Picking System. This system is an automated single-cell isolation system that allows the isolation of rare cells from a large number of candidate cells via the analysis of immunofluorescence signals. This is the first report indicating that the new cell picking system can be used to isolate CTCs in clinical samples. We performed a comparative analysis of cells obtained using the CellSearch® system and the single-cell picking system. The new system resulted in 26.6% cell loss, on average, relative to the number of cells obtained using the CellSearch® system. The lower cell counts may reflect manual processing issues, such as pipetting errors. The re-identification rate observed using the single-cell picking system is comparable to that of another previously reported device, the DEPArray™ system (Silicon Biosystems, Bologna, Italy) [26, 27]. The recovery rate in a small number of cells was 70.8%, on average (range 38.5–100%). This result demonstrated the feasibility of this application in a more clinically relevant range.

In a preclinical validation of the *KRAS* mutation analysis of single cells, known mutations were confirmed in 87.5% of samples. The other 12.5% of samples showed the wild-type allele, which may indicate contamination with normal cells during CTC selection. In this examination, no wild-type *KRAS* cells were found. Thus, loss of mutant-type allele was not occurred and false negativity was not detected. This result indicated that the system is feasible for the detection of *KRAS* mutations by liquid biopsy.

In our study, we demonstrated analyses of *KRAS* mutations using two different DNA amplification methods, direct PCR and WGA. We showed the feasibility of *KRAS* mutation analyses using both methods. Direct PCR is more convenient with respect to time and cost compared with WGA, but few mutations can be analyzed. If information for a single mutation is needed (i.e., EGFR T790 M for targeted therapy in lung cancer) for treatment choices, direct PCR might be suitable. WGA can be used for multi-locus molecular profiling. In the colorectal cancer field, information for several mutations is required for treatment decisions, therefore the WGA method is appropriate.

We analyzed *KRAS* mutations in single CTCs and matched primary tumors from patients with mCRC. In total, 36.4% of patients had *KRAS* mutations in CTCs, whereas 27.3% of patients had mutations in primary tumors. In 10 of 11 (90.9%) cases, the *KRAS* status of the primary tumor matched that of CTCs by either direct PCR or WGA methods. In one patient (Patient IX, Table 4), we found discordant results between the *KRAS* status of single CTCs and the primary tumor. In this case, the mutation was found in the CTC and wild-type *KRAS* was found in the primary tumor. The mutation may be present in only a minor subclone of the primary tumor. Although a number of reports have examined the concordance between *KRAS* mutations in primary tumors and metastatic lesions in mCRC, the significance of observed cases of discordance has only recently been considered [28–30]. Several studies have shown discrepancies between the genetic profiles of CTCs and primary tumors [31, 32] and heterogeneity among individual CTCs [27]. Because single-CTC analyses by liquid biopsy provide information regarding the real-time status of existing tumors, these data might provide more accurate information for personalized therapy.

In one patient (Patient IX), *KRAS* mutations in CTCs differed among blood samples obtained at different time periods. One CTC had a p.G13D mutation, and the other had p.G12D. In another patient (Patient III), the mutation was homozygous in one CTC, but heterozygous in another CTC. In these cases, either more than one subclone was present in a tumor at a given time or a mutation was acquired during the clinical course of the disease. These results are consistent with the growing number of studies reporting high heterogeneity among CTCs within a patient [18, 33–35]. Our results raise several clinical questions about the real value and significance of CTC analyses. One question is which status is appropriate for treatment decisions if the CTC mutational status was different from that of the primary tumor. Another question is which mutational status is the most clinically significant if CTCs show genetic heterogeneity. Although heterogeneity among single CTCs has been observed at several loci that are drug targets (e.g., EGF receptor inhibitors) or associated with drug resistance (e.g., PIK3CA and KRAS), the clinical relevance of this variation is unknown. To address these questions, clinical studies are needed to monitor changes in the mutational status of CTCs and primary and/or metastatic tumors during treatment as well as to identify indicators of the treatment response.

## Conclusions

We examined the molecular profiles of single CTCs using the ASONECell Picking System, a new cell sorter that enables the isolation of single or small groups of cells from mixed-cell suspensions. We demonstrated that the isolation and molecular characterization of single CTCs is feasible in mCRC patients. We detected CTC heterozygosity as well as differences between primary tumors and CTCs with respect to *KRAS* status. This system may facilitate future analyses of the clinical significance of CTC heterogeneity.

## Abbreviations

APC: Allophycocyanin
CRC: Colorectal cancer
CTC: Circulating tumor cell
HD: Healthy donor
mCRC: metastatic colorectal cancer
PE: Phycoerythrin
WGA: Whole-genome amplification
